# Advances in Parasite Genomics: From Sequences to Regulatory Networks

**DOI:** 10.1371/journal.ppat.1000649

**Published:** 2009-10-30

**Authors:** Elizabeth A. Winzeler

**Affiliations:** Department of Cell Biology ICND202, The Scripps Research Institute, La Jolla, California, United States of America; University of California San Francisco, United States of America

Parasites have kept many secrets from the researchers who have sought to eradicate them over past decades. The mechanisms by which they evade drugs, escape the immune system, regulate switching between genes involved in immune evasion, and orchestrate development have been difficult to elucidate. They have been successful at this in part because they are difficult to keep in the laboratory, difficult to breed, and difficult to raise in sufficient quantities for biochemistry, and because they parasitize hosts that are not ideal experimental subjects. While *Plasmodium falciparum* is less tractable than one would wish, genetic manipulation can still be performed. On the other hand, *Plasmodium vivax*, which cannot be maintained in culture, is even less accessible, and there are few research tools available.

While these difficulties present impediments to drug, vaccine, and basic research, the availability of parasite genome sequences and related genome-based tools have provided substantial opportunities to overcome the lack of a robust culture system needed for traditional molecular biology, the shortage of material for biochemistry, and the lack of traditional genetic methods for studying gene function. The advent of new technologies for examining and detecting genetic variation, measuring transcript abundance, and measuring protein or metabolite abundance on a genome-wide scale, or for sequencing genomes in combination with new computational methods, may lift some of the barriers to working on actual pathogens. Here, I will review some recent discoveries that were facilitated by industrial-scale molecular biology approaches.

## New Genome Sequences

The year 2008 witnessed the publication of the complete genome sequence of *P. vivax* as well as that of *Plasmodium knowlesi* [1],[2]. Although *P. vivax* may be responsible for up to 40% of the 515 million malaria cases each year, work on this parasite has generally lagged because it cannot yet be maintained in long-term culture. Among the highlights of the *P. vivax* genome sequence was the observation that it encodes a variety of cell-binding proteins involved in erythrocyte selection, and thus *P. vivax* may be able to use a variety of red cell invasion strategies. Of course, knowing the complement of genes encoded by a genome only serves as a prelude to further functional studies, and the first set of gene expression data for *P. vivax* was published soon afterwards [3]. This work showed that the transcriptional program of *P. vivax* is similar to that of *P. falciparum* and offers hypotheses about the function of a variety of *P. vivax* genes. For example, a gene whose transcriptional pattern is correlated with those of known invasion genes may also be involved in invasion. Accompanying the publication on the *P. vivax* genome was the sequence of *P. knowlesi*, described as the fifth human pathogen given its documented zoonoses [4]. This genome sequence reveals intriguing examples of molecular mimicry [2]. It was shown that members of the multigene family encoding the KIR proteins have a predicted extracellular domain that shows stretches of identity to host proteins with particularly strong matches to CD99, a human immunoregulatory protein found on the surface of T cells and other lymphocytes. These data raise the interesting possibility that the *kir* gene products may play a more active role in immune suppression through competition with T cells for CD99 partner molecules rather than just functioning as an antigenic smokescreen, a presumed role for many of the proteins encoded by highly variable *Plasmodium* multigene families (*vars*, *stevors*, *virs*).

## Genetic Regulatory Networks

In organisms that are relatively difficult to genetically manipulate, genomic methods offer opportunities to define regulatory networks by linking motifs in the promoters of co-expressed genes to the DNA-binding activity of different transcription factors. It was recently shown that sets of co-transcribed genes in *P. falciparum* often share short sequence motifs upstream of their ATGs at rates not expected by chance [5]. A similar approach has been shown to work in *Toxoplasma gondii*, where functional annotations served as a substitute for gene expression groupings [10]. Although site-directed mutagenesis in *P. falciparum* has validated the importance of some of these motifs controlling promoter activity, the identity of proteins that bind these motifs has remained generally obscure. However, recently de Silva and coworkers used a protein-binding microarray that contains every possible 10-mer [6] to discover the motifs bound by a series Apicomplexan AP2 transcription factors [7]. These are members of a putative transcription factor family discovered by bioinformatic searches and are homologous to a family in *Arabidopsis* named the AP2/ERF DNA-binding family [8]. Remarkably, several of these motifs were near perfect matches to the set of motifs shown to be associated with genes involved in invasion or exoerythrocytic stage function in the transcriptional analysis [5]. Moreover, Yuda and coworkers provided genetic confirmation that one of the AP2 proteins regulates genes expressed in the ookinete stage [9] by binding to specific six-base sequences in the proximal promoter. The next challenge will be to perform chromatin immunoprecipitation studies on all DNA-binding proteins and to examine their genome-wide occupancy with a goal of creating a complete map.

## Epigenetics of Antigenic Variation

While specific promoter elements are likely to regulate some genes, chromatin structure may play a major role in controlling transcription of genes involved in antigenic variation in multiple parasite species. Malaria parasites and trypanosomes both have large sets of genes that are involved in antigenic variation, and while the two species are well separated on the tree of life, epigenetics appear to control expression of genes involved in antigenic variation in both species. In African trypanosomes, it was shown that a particular histone methylase is responsible in repressing variant surface glycoprotein genes involved in antigenic variation [11]. In malaria parasites, disrupting the histone deacetylase PfSir2A, but not PfSir2B, also results in derepression of genes involved in antigenic variation [12]. Genome-wide chromatin immunoprecipitation studies have also shown correlations between various histone modifications [13],[14], or *P. falciparum* heterochromatin protein 1 [15] and the location of clonally variant gene families in *P. falciparum*. Likewise, histone variants mark the start of polycistronic Pol II transcripts in trypanosomes [16]. The patterns of histone occupancy and modification may lead to new theories for how the regulation and switching of antigenic variation genes, critical to pathogenesis, are controlled.

## Expression Quantitative Trait Loci

**Figure 1:**
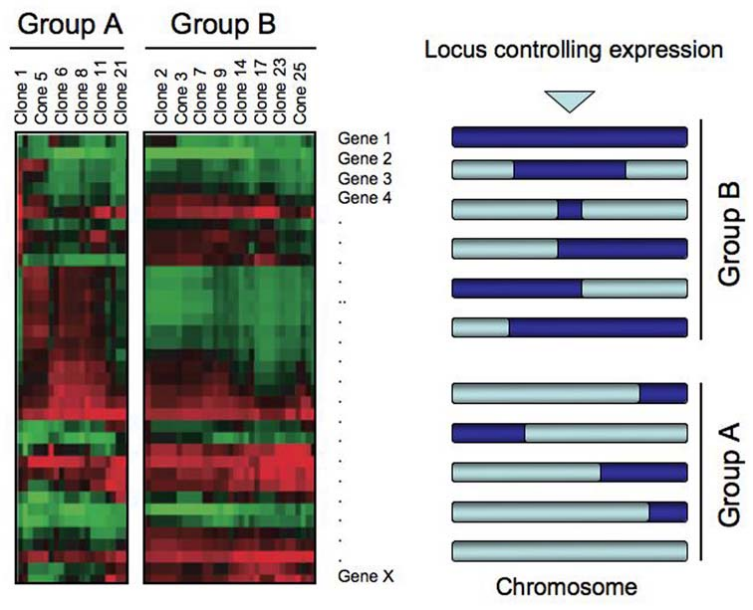
Expression quantitative locus (eQTL) mapping. In this method, different clones from a genetic cross are expression profiled, potentially resulting in two or more different groups, which show distinct expression patterns for a variety of genes as represented by the red-green heat map. Genotyping is then performed on the clones. Loci are identified that are shared by all the clones with the group A pattern, but not by clones with the group B pattern (hypothetical data). The locus may encode a transcriptional regulatory protein that controls the expression of a variety of different genes.

Sexual crosses can be difficult to perform in parasites. Nevertheless, laboratory crosses have been performed on several occasions for *T. gondii* and *P. falciparum*. The resulting progeny have been used to map genes involved in drug resistance, host specificity [3], and virulence. While the crosses were usually set up to map a particular trait (e.g., chloroquine resistance), the progeny strains can also be used to map the locus responsible for any quantitative phenotypic difference that separates the two parental lines. Such phenotypes may include growth rate, host cell invasion pattern, differences in the immunolocalization pattern of a given marker, or even gene expression differences [17] that are mapped using a method called expression quantitative trait locus (eQTL) mapping. eQTL work involves the use of linkage mapping to locate genome regions that determine transcript abundance. Both *cis* loci and *trans* loci can be identified. An allele that gives rise to a *cis* eQTL might affect transcript abundance for just that gene by affecting promoter activity or transcript degradation rates, while a *trans* eQTL, potentially in a transcription factor or an RNA-binding protein, might affect the transcript levels at a variety of unlinked loci. By examining the full genome expression profile of different progeny from a genetic cross, one can determine potential regulatory loci shared by all strains having the same expression phenotype (Figure 1). In *P. falciparum*, expression studies were performed on a series of progeny clones from a genetic cross between a chloroquine-resistant strain (Dd2) and chloroquine-sensitive strain (HB3) [18]. The authors of this paper identified a powerful *trans* eQTL on chromosome 5 that controls expression at a large number of genes across the genome and co-localizes with an important drug resistance gene (*pfmdr1*). However, similar studies using the progeny of a genetic cross between a virulent and less virulent strain of *T. gondii* only revealed *cis*-acting loci, indicating that virulence differences were likely to be in polymorphic genes [19] and not in any regulatory factor. Because different host strains are known to be more or less susceptible to parasite infection, the same approach could be used to map regulatory genes controlling the host's response to infection by examining expression profiles of white blood cells or in affected organs in susceptible and nonsusceptible hosts.

## Translating Genomics into Drug Discovery

Over the past several years, the problem of rapidly emerging drug resistance has led to substantial investments in drug discovery programs that have sought to place new drugs for neglected diseases into the pipeline. Drug discovery efforts have benefited from genome sequencing programs that have revealed targets that are found in parasites but are lacking in humans. However, an additional and potentially unrecognized benefit of having parasite genome sequences is that they offer a very powerful approach for rapidly determining an uncharacterized drug's likely mechanism of action or target using *in vitro* evolution studies. This classic method, which involves growing microbes in sub-lethal concentrations of a drug until they become resistant and then mapping the mutant allele through complementation, has been available to bacteriologists for many years. Because parasites may lack efficient complementation methods, parasitologists have had to wait for the advent of full genome sequencing or the availability of comprehensive full genome tiling arrays to use this approach. Recently, Dharia et al. showed that tiling microarrays, in addition to uses in discovering new transcripts [20] or characterizing variation [21], could be used to detect a copy number variant responsible for fosmidomycin resistance and a newly emerged point mutation responsible for blasticidin resistance [22]. Full genome deep sequencing methods also may give similar results and may be the only option for diploid organisms. Copy number variants or SNPs discovered in the laboratory and associated with drug resistance may eventually be examined in the field. Nair et al. examined linkage disequilibrium with a previously identified copy number variant and showed that GTP cyclohydrolase I amplifications are in linkage disequilibrium with key drug resistance mutations in dihydrofolate reductase [23], suggesting a functional linkage between these two genes.

The frontier of parasite genomics is probably not in sequencing more parasite species or in collecting gene expression data from another pair of conditions. Advances are more likely to be through the integration of large multifaceted datasets, and through studies of complex systems, such as the global transcriptome of the parasite in immune and nonimmune patients, or susceptible and nonsusceptible inbred mice lines. In addition, there are great opportunities for combining population biology with genomics. One could imagine in the future pinpointing the molecular basis of drug resistance through eQTL mapping using expression profiles of parasites obtained from the blood of individuals who had clinically failed treatment. Before this can be realized, however, similar advances in methods for phenotyping parasites will need to be developed. Nevertheless, it seems likely the impact of genomics will soon be measured at the bedside.
